# Stress, coping and adherence to immunosuppressive medications in kidney transplantation: a comparative study: Estresse, *coping* e aderência a medicamentos imunossupressores em transplante renal: um estudo comparativo

**DOI:** 10.1590/1516-3180.2015.01071008

**Published:** 2015-12-08

**Authors:** Daniela Cristina Sampaio de Brito, Elisa Oliveira Marsicano, Fabiane Rossi dos Santos Grincenkov, Fernando Antônio Basile Colugnati, Giancarlo Lucchetti, Helady Sanders-Pinheiro

**Affiliations:** I MSc. Attending Psychologist, Renal Transplantation Unit, Division of Nephrology, Federal University of Juiz de Fora, and Research Fellow, Núcleo Interdisciplinar de Estudos e Pesquisas em Nefrologia (NIEPEN), Juiz de Fora, Minas Gerais, Brazil.; II MSc. Assistant Professor of Nursing, Renal Transplantation Unit, Division of Nephrology, Federal University of Juiz de Fora, and Research Fellow, Núcleo Interdisciplinar de Estudos e Pesquisas em Nefrologia (NIEPEN), Juiz de Fora, Minas Gerais, Brazil.; III PhD. Adjunct Professor of Psychology, Renal Transplantation Unit, Division of Nephrology, Federal University of Juiz de Fora, and Research Fellow, Núcleo Interdisciplinar de Estudos e Pesquisas em Nefrologia (NIEPEN), Juiz de Fora, Minas Gerais, Brazil.; IV MD, PhD. Adjunct Professor of Medicine, Renal Transplantation Unit, Division of Nephrology, Federal University of Juiz de Fora, and Research Fellow, Núcleo Interdisciplinar de Estudos e Pesquisas em Nefrologia (NIEPEN), Juiz de Fora, Minas Gerais, Brazil.; V MD, PhD. Adjunct Professor of Medicine, Department of Medicine, Núcleo de Pesquisas em Espiritualidade e Saúde (NUPES), Federal University of Juiz de Fora, Brazil.; VI MD, PhD. Associate Professor of Medicine, Head of Renal Transplantation Unit, Renal Transplantation Unit, Division of Nephrology, Federal University of Juiz de Fora, and Research Fellow, Núcleo Interdisciplinar de Estudos e Pesquisas em Nefrologia (NIEPEN), Juiz de Fora, Minas Gerais, Brazil.

**Keywords:** Stress, psychological, Adaptation, psychological, Medication adherence, Patient compliance, Kidney transplantation, Estresse psicológico, Adaptação psicológica, Adesão à medicação, Cooperação do paciente, Transplante de rim

## Abstract

**CONTEXT AND OBJECTIVE:**

Adherence to medication is a key issue relating to outcomes from transplantation and it is influenced by several factors, such as stress and coping strategies. However, these factors have been poorly explored. We aimed to compare stress and coping strategies between adherent and nonadherent renal transplant recipients who were receiving immunosuppression.

**DESIGN AND SETTING:**

We conducted a comparative, cross-sectional and observational study at a university-based transplantation clinic in Juiz de Fora, Brazil.

**METHODS:**

Fifty patients were recruited and classified as adherent or nonadherent following administration of the Basel Assessment of Adherence to Immunosuppressive Medications Scale. Stress was evaluated using the Lipp Stress Symptom Inventory for Adults and coping strategies were assessed using the Ways of Coping Scale.

**RESULTS:**

The study included 25 nonadherent patients and 25 controls with a mean age of 44.1 ± 12.8 years and median post-transplantation time of 71.8 months. Stress was present in 50% of the patients. Through simple logistic regression, nonadherence was correlated with palliative coping (OR 3.4; CI: 1.02-11.47; P < 0.05) and had a marginal trend toward significance with more advanced phases of stress (OR 4.7; CI: 0.99-22.51; P = 0.053).

**CONCLUSION:**

Stress and coping strategies may have implications for understanding and managing nonadherent behavior among transplantation patients and should be considered among the strategies for reducing nonadherence.

## RESUMO

**CONTEXTO E OBJETIVO:**

Aderência à medicação é uma questão chave para o resultado do transplante e é influenciada por diversos fatores, tais como o estresse e estratégias de enfrentamento ou *coping*. Entretanto, esses aspectos têm sido pouco explorados. Compararmos o estresse e as estratégias de *coping* em paciente transplantados renais, aderentes e não aderentes, em uso de imunossupressores.

**TIPO DE ESTUDO E LOCAL:**

Realizamos estudo comparativo, transversal e observacional em uma clínica universitária de transplantes em Juiz de Fora, Brasil.

**MÉTODO:**

Cinquenta pacientes foram selecionados e classificados como aderentes e não aderentes a partir da escala Basel Assessment of Adherence to Immunosuppressive Medications Scale. O estresse foi avaliado pelo Inventário de Sintomas de Estresse para Adulto de Lipp e as estratégias de *coping* foram avaliadas pela escala Escala de Modos de Enfrentamento de Problemas.

**RESULTADOS:**

O estudo inclui 25 pacientes não aderentes e 25 controles com idade média de 44,1 ± 12,8 anos e mediana de tempo de transplante de 71,8 meses. Estresse esteve presente em 50% dos pacientes. Por regressão linear simples, a não aderência foi associada com o *coping* paliativo (OR 3,4, CI: 1,02-11,47; P < 0,05) e teve uma tendência marginal a significância com as fases mais avançadas do estresse (OR 4,7, CI: 0,99-22,51; P = 0,053).

**CONCLUSÃO:**

Estresse e estratégias de *coping* podem trazer implicações na compreensão e manejo do comportamento de não aderência dos pacientes transplantados e deveriam ser considerados nas estratégias na redução da não aderência.

## INTRODUCTION

Kidney transplantation is associated with higher survival rates, better quality of life and fewer public health costs than those of dialysis programs.^1^^,^^2^ These outcomes have been achieved mainly because of the use of immunosuppressive therapy.^3^ Nonetheless, long-term survival has not improved to the same degree, and this has therefore become a great challenge for healthcare providers.^4^

According to several studies, strict adherence to the drug regimen is one of the main goals of efficient treatment, and this reduces the frequency of complications, such as late acute rejection episodes and late graft loss.^5^^,^^6^ Adherence is defined as “the degree to which a person’s behavior corresponds to the recommendations from a healthcare provider”.^7^ This concept is influenced by several factors.^5^^,^^7^^,^^8^^,^^9^ One potential theory that could lead to attainment of this multilevel interaction is the Ecological Model. This model maintains that behavior that interferes with adherence is a result of interaction between factors at multiple levels. These different levels can be divided into “the patient” and the “micro”, “meso” and “macro” levels. Specific characteristics of the individual, like psychiatric disorders, stress and coping strategies, are included at the patient level.^10^^,^^11^

On the other hand, nonadherence in the field of transplantation, defined as any deviation from the drug regimen prescribed that negatively affects the results,^12^ represents risky behavior and is associated with reduced kidney allograft survival, lower quality of life and increased public spending.^5^^,^^6^^,^^13^^,^^14^ Unfortunately, nonadherence to immunosuppressants is common among kidney transplantation patients and some reports have shown that kidney recipients are the most nonadherent among all transplantation patients.^6^^,^^15^^,^^16^

Within this context, since adherence is multifactorial and is related to socioeconomic, individual, clinical and healthcare system variables,^7^^,^^11^ exploration of which individual factors can have an influence on nonadherence is needed. Although many psychological dimensions contribute towards nonadherence, only a few of them have been extensively studied.^17^ Among all of these factors, particular attention should be given to mental health (depression and anxiety), stress and coping patterns.^17^^,^^18^

Stress, as was first described by Seyle,^19^ can be defined as an organism’s response to challenging events. It may also be understood as the relationship between the individual and the environment. There is a clear association between high and persistent levels of stress and the onset or worsening of several chronic pathological conditions.^20^^,^^21^ Despite the well-established benefits of kidney transplantation, it does not eliminate all health-related stress.^22^^,^^23^^,^^24^^,^^25^^,^^26^^,^^27^^,^^28^ Many challenges are faced after kidney transplantation, such as following a complex medication regimen, dealing with its side effects, living constantly under the influence of feelings of uncertainty or fear relating to graft survival, and the social pressure to return to the previous routine.^22^^,^^23^^,^^24^^,^^25^^,^^26^^,^^27^^,^^28^

Another important aspect in this complex interaction is how patients cope with their condition. Coping refers to a set of cognitive and behavioral efforts aimed at controlling, reducing or eliminating stress.^29^ These strategies may be classified according to function: coping focused on the problem (trying to modify the stressor); and coping focused on emotion (trying to regulate the emotional response to stress).^29^ Coping patterns contribute towards management of kidney transplantation-related stressors and maintenance of quality of life.^17^ However, certain strategies may lead to ineffective adaptation to the demands of the illness and the treatment. Emotion-focused strategies have been correlated with more frequent recognition of stressors and less perception of stress control among kidney transplantation patients.^30^

Therefore, identification of potentially modifiable variables, like stress and coping patterns, could improve adherence behavior relating to medications, and consequently, the clinical outcomes from kidney transplantation.^8^

## OBJECTIVE

The present study aimed to compare coping strategies and stress between adherent and nonadherent kidney transplantation patients receiving immunosuppression.

## METHODS

### Design

We conducted a single-center comparative, cross-sectional and observational study at a university-based transplantation clinic located in the city of Juiz de Fora, Brazil (Núcleo Interdisciplinar de Estudos, Pesquisas e Tratamento em Nefrologia, NIEPEN) between August and December 2010.

### Sample and setting

The study sample was recruited from a previous study dealing with validation of the Basel Assessment of Adherence to Immunosuppressive Medication Scale (BAASIS) for use in Portuguese.^31^ Patients were included based on the following criteria: age of at least 18 years, more than one year after transplantation, and willing to participate in the study; which resulted in a convenience sample of the first 100 kidney transplantation patients who were being followed up at our outpatient facility.

All the patients answered the BAASIS questionnaire, which is a transculturally adapted self-reporting instrument developed by the Leuven-Basel Adherence Research Group, in Basel, Switzerland,^32^^,^^33^ and which has been validated by our research team.^31^ BAASIS was administered during the patients’ regular consultation visits, by transplantation nurses who had been trained as interviewers. However, the patients did not receive any specific feedback about their adherence status during their responses to the questionnaire.

Then, from August to December 2010, all patients routinely scheduled for medical consultations and included in our previous validation study^31^ were consequently invited to participate in this new study. Patients were included if they had received their transplant more than one year earlier (thus denoting that stable graft function had been achieved) and were at least 18 years old. No patients with retransplantation or who were unable to understand the objectives of the study or had difficulties in filling out the questionnaire were included.

**Figure 1.**
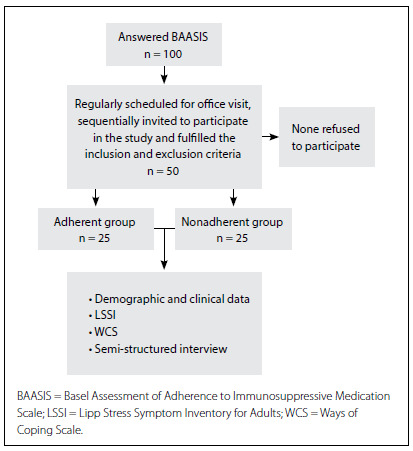
Study design.

The first 25 adherent patients (adherent group) and the first 25 nonadherent patients (nonadherent group) were selected, totaling 50 patients (Figure 1). None of the first 50 invited patients who fulfilled the inclusion and exclusion criteria declined to participate.

### Variables and measurements

#### General data

Demographic and clinical data were collected through retrieving the following information from the medical records: gender, age, marital status, years of formal education, city of origin, type of donor, time of kidney transplantation, serum creatinine and comorbidities.

### Adherence

The definition of nonadherence that was used was based on the medication regimen as recommended by the transplantation community.^12^ As previously mentioned, to evaluate adherence to immunosuppressive drugs, we applied BAASIS,^32^^,^^33^^,^^34^ as validated for use in Portuguese.^31^ BAASIS assesses relevant dimensions of immunosuppressive drug use (i.e. adherence to taking the drug, adherence to the times for taking the drug, drug holidays and dose reduction) over a fixed time period consisting of the last four weeks. Responses are given on a six-point scale: never (0), once per month (1), every second week (2), every week (3), more than once per week (4) and every day (5). Any deviation, namely an answer differing from “never,” among any of the items, is considered to be nonadherence. Cronbach’s alpha for the validated translation into Portuguese was 0.70, thus indicating moderate internal consistency.^31^

### Stress

The presence and level of stress were evaluated using the Lipp Stress Symptoms Inventory (LSSI) for adults.^35^ The LSSI comprises a questionnaire that detects the presence of stress and classifies patients in accordance with Lipp’s four-phase model (alert, resistance, quasi-exhaustion and exhaustion). We opted to use LSSI because it was developed and validated in Portuguese (Cronbach’s alpha of 0.91).^35^ We first evaluated the presence of stress, then used the original stress phases and finally used a composite classification that included an initial stress phase (patients in the alert and resistance phases) (initial phase) and a more advanced phase (patients in the quasi-exhaustion and exhaustion phases) (more advanced phase).^35^

### Coping

Coping was assessed using the WCS, a 45-item scale developed by Vitaliano et al.^36^ and validated for use in Portuguese.^37^ The items on this self-reporting instrument are rated on a five-point Likert scale: I never do this (1); I do this a little bit (2); I sometimes do this (3); I do this a lot (4); and I always do this (5). Four patterns of coping were evaluated: coping focused on the problem (18 items; Cronbach’s alpha: 0.84); coping focused on emotion (15 items; Cronbach’s alpha: 0.81); searching for/turning to religion/fantasy thoughts (7 items; Cronbach’s alpha: 0.74); and seeking social support (5 items; Cronbach’s alpha: 0.70).^37^ Higher scores indicate greater use of each coping strategy. A further analysis was also performed, in which these original four categories were grouped into two others: active coping, focused on the problem and seeking social support; and palliative coping, focused on emotion and searching for/ turning to religion/fantasy thoughts. This categorization was based on the general goal of the coping response, which may be directed towards the situation (active coping) or towards emotional management (palliative coping).^29^

### Data collection

First, we invited outpatients who fulfilled the study inclusion and exclusion criteria. As mentioned previously, BAASIS had been administered to these patients during their previous regular consultation visit by trained transplantation nurse interviewers. Then, participants were given written and oral information before signing the informed consent form. Finally, the LSSI and the WCS were applied by a psychologist from outside the kidney transplantation team. The time interval between applying BAASIS and applying LSSI and WCS was about a month, and the data were gathered from all of these questionnaires between August and December 2010. Complementary data were gathered from the medical records.

### Ethical considerations

This study was approved by the local research ethics committee (approval number 0028/2010). Patients agreed to participate in the study through signing an informed consent form.

### Statistical procedures

Descriptive statistical analyses were conducted using frequencies to evaluate categorical variables and means and standard deviations to evaluate continuous variables. The t test, Mann-Whitney test and chi-square or Fisher’s test were used to compare variables between the adherent and nonadherent groups (Tables 1 and 2). Odds ratios were then estimated by means of simple logistic regression on stress and coping relating to nonadherence. We analyzed stress in three categories: no stress, initial stress phase and more advanced stress phase. For coping, we considered two grouped categories for the analysis: palliative coping and active coping. A multivariate approach was not feasible due to the small sample size and homogeneity of the adherent and nonadherent groups, as shown in the results section. We presented odds ratio point estimates and their respective 95% confidence intervals.^38^^,^^39^ The significance level was set at 0.05. The analyses were performed using the SPSS 15.0 statistical package (SPSS Inc., Chicago, IL, USA).

**Table 1.**
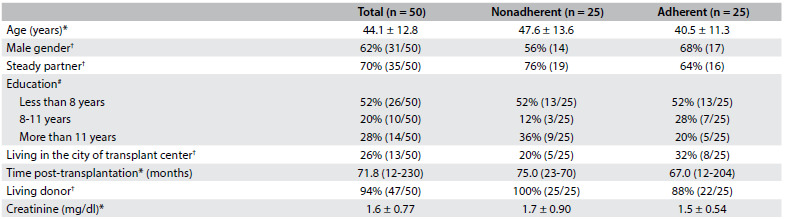
Demographic and clinical variables of adherent and nonadherent kidney transplantation patients studied

Continuous variables * were compared using t test or Mann-Whitney test, and frequencies ^†^ using chi-square test. No significant differences were found between the groups.

**Table 2.**
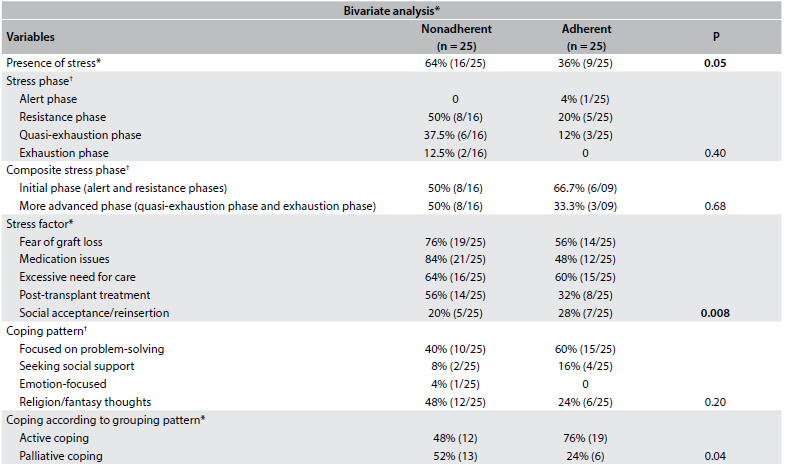
Bivariate analysis on the presence of stress, stress factors and coping patterns among adherent and non-adherent kidney transplantation patients

*Variables were analyzed using chi-square * or Fisher test ^†^.

## RESULTS

### Descriptive data

The patients’ mean age was 44.1 ± 12.8 years, and 62% were male. Ninety-four percent received their graft from a living donor. The median post-transplantation time was 71.8 months (range: 12-230), and the creatinine level was 1.6 ± 0.74 mg/dl. Fifty-two percent of the patients had finished primary school, 20% secondary school and 28% higher education. Most of the individuals (70%) reported having a steady partner, and only 26% lived in the city of the transplantation center. No significant differences were found in the descriptive data between the adherent and nonadherent groups (Table 1).

### Stress and adherence

According to the LSSI, 50% of the patients had a diagnosis of stress. However, we found higher frequency of stress in the nonadherent group (64%) than in the adherent group (36%) (P = 0.05) (Table 2). On the other hand, neither of the groups showed any differences between the stress phases (P = 0.40) and the composite stress phases (P = 0.68).

Likewise, we did not find any factor independently associated with nonadherence in bivariate analysis (Tables 1 and 2), including demographic and clinical data. Because stress and coping variables behaved collinearly, a simple logistic regression model was used to estimate odds ratios and their respective 95% confidence intervals (CI) for stress levels and coping pattern, in relation to adherence. The more advanced level of stress showed a nonsignificant trend towards a positive association with nonadherence to immunosuppressive medication in this analysis (OR 4.7; 95% CI: 0.99-22.51; P = 0.053) (Table 2).

### Coping patterns and adherence

There was a difference in the coping patterns relating to searching for religious practices and fantasy thoughts between the groups (52 versus 24%), but the results were not statistically significant (Table 2). In contrast, after grouping the coping patterns, adherent patients had more responses associated with active coping directed towards the stressor (76%) than did nonadherent patients (48%) (P = 0.04) (Table 2). Moreover, the palliative coping pattern (religion/fantasy thoughts and emotion-focused coping) was associated with nonadherence (OR 3.4; 95% CI: 1.02-11.47) (Table 3).

**Table 3.**
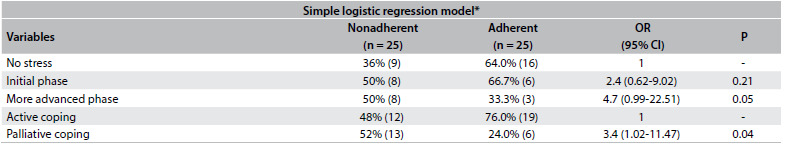
Simple logistic regression model for the presence of stress, stress factors and coping patterns among adherent and non-adherent kidney transplantation patients

*Logistic regression model. We ran two different logistic regression models, one for stress and the other for coping pattern.

## DISCUSSION

In this comparative study, we found that nonadherent patients had high stress (50%) and used more palliative coping strategies than did adherent patients. Although the patients had been previously grouped according to their adherence to immunosuppressive drugs, the demographic and clinical variables were similar between the adherent and nonadherent groups.

These results appear consistent with the conclusions from previous studies, in which kidney transplantation did not eliminate chronic kidney disease-related stressors and those relating to disease treatment, since kidney transplantation is only a form of therapy for chronic kidney disease, and not its cure.^2^ Even with a well-functioning graft, these individuals continue to be chronic disease patients who are subject to some level of social, physical and emotional limitations, as is the case with dialysis patients.^9^^,^^22^^.^^23^^,^^24^^,^^25^ However, differently from our study, most other studies failed to directly assess the frequency of stress.

In order to assess stress, we used the LSSI, which is a psychological test that enables diagnosing of stress based on specific symptoms and identification of the stress phase, in accordance with Seyle’s theory.^19^ This is the only instrument for evaluating stress that has been validated for use among Portuguese-speaking patients and that has characteristics in line with the aims of our study. Nevertheless, previous studies evaluating stress and kidney transplantation have used other instruments such as scales based on subjective measurement of stressful factors or specific life themes, including those relating to the illness.^22^^,^^23^^,^^24^^,^^25^^,^^28^ Those scales probably have limited evaluation capacity because they rely on patient memory and do not consider the coping strategies used by patients, which may alter the stress results.^35^

Additionally, we did not find any studies that have shown any consistent link between the specific phases of stress and nonadaptive behavior of nonadherence in cases of kidney transplantation. Stressed patients, particularly those in the advanced phase, are more likely to develop emotional problems (depression and anxiety), cognitive problems (attention and memory) and affective problems (interpersonal conflicts or social withdrawal).^35^ Each of these conditions may potentially contribute towards ineffective coping with health status, thus affecting adherence to medication among kidney transplantation patients.

Concerning coping patterns, nonadherence to immunosuppressants was associated with palliative control in our study, mainly focused on “emotion” and “religion/fantasy thoughts”. Coping responses “focused on the problem” or “focused on emotion” are fundamental for attenuating the impact of stressors, given that both of these responses play complementary yet different roles in stress control.^40^ However, over a long period of time, active coping strategies tend to be more adaptive, because they attempt to confront the stressor, thereby reducing stress-related symptoms and helping achieve an adjustment to the stressor situation.^41^ We have already identified the most frequent stressors in our population.^42^ Strategies to help this population deal with the stressors, designed individually or generally, are now the focus of our attention. We have reinforced our educational activities in waiting room and have taken an interdisciplinary approach towards better social support, with psychotherapy for cognitive restructuring and use of motivational interview techniques.

Thus, in the context of adherence to medication among kidney transplantation patients, we expected palliative coping to be related to nonadaptive nonadherence behavior. In fact, Lindqvist et al. observed that patients who used more evasive, fatalistic, palliative and emotional coping strategies were less capable of dealing with the demands of chronic kidney disease and kidney transplantation.^30^ Another study on a sample comprising 200 kidney transplantation patients showed that recipients who reported higher stress and more depression, and who coped with stress by using avoidant coping strategies, were less compliant with medication.^41^

There are some limitations to our study. The sample size, similarly to other studies involving psychological factors among kidney transplantation patients,^9^^,^^17^ was less than 100 patients. We tried to overcome this limitation by applying a sampling design that involved studying the same numbers of adherent and nonadherent subjects, selected from the main study population (BAASIS validation).^31^ However, we recognize that this design was underpowered, compared with case-control matched studies. Use of a single self-reporting instrument may have limited the evaluation of immunosuppressive adherence.

We opted for BAASIS as the method for assessing adherence because it was one of the three measurement tools for self-reporting of nonadherence that the Transplant 360 Task Force identified as presenting the potential for effective adaptation for use in transplantation clinical practice.^32^^,^^33^ Moreover, BAASIS is the only instrument that has been validated for use among Brazilian Portuguese-speaking transplantation patients.^31^

In addition, living donor recipients predominated in our sample. Until recently, this donor profile was the most common type of transplantation performed in Brazil.^43^ Future studies should include patients receiving transplants from deceased donors, in order to enable greater understanding of the issues relating to this condition.

The fact that each patient was at a specific post-transplantation time prevented investigation of the potential association between stress and coping over the entire transplantation experience. We also take the view that, because of the design of the present study, it was not possible to evaluated causality and whether stress and coping were the cause or consequence of nonadherence. Moreover, since multivariate analysis was not possible, clinical inferences should be made with caution.

Nevertheless, our results present relevant results, given the paucity of studies in this field and the absence of previous studies among the Brazilian population. A longitudinal follow-up study, including key transplantation periods (i.e. pre, peri and post-transplantation stages), could provide additional evidence with regard to these questions.

## CONCLUSIONS

The present study showed that stress occurs frequently, even in cases of well-functioning kidney transplantation. Palliative patterns of coping with transplant-related stressors were independently associated with immunosuppressive nonadherence. These findings support the notion that adherence interventions should integrate behavioral, psychosocial and medical approaches in order to appropriately limit the undesirable consequences of nonadherence in kidney transplantation. Therefore, more studies are needed in this field of research. Healthcare professionals should be prepared to provide whole-person care for their transplantation patients, taking into consideration their bio-psycho-social-spiritual needs and proposing interventions for improving adherence behavior relating to medication and, consequently, the outcomes from kidney transplantation.
